# Retrospective analysis of recurrence patterns and clinical outcome of grade II meningiomas following postoperative radiotherapy

**DOI:** 10.1186/s13014-021-01825-2

**Published:** 2021-06-25

**Authors:** Elgin Hoffmann, Kerstin Clasen, Bettina Frey, Jakob Ehlers, Felix Behling, Marco Skardelly, Benjamin Bender, Jens Schittenhelm, Matthias Reimold, Ghazaleh Tabatabai, Daniel Zips, Franziska Eckert, Frank Paulsen

**Affiliations:** 1 Department of Radiation Oncology, University Hospital Tuebingen, Hoppe-Seyler-Str. 3, 72076 Tuebingen, Germany; 2 Center of Neuro-Oncology, Comprehensive Cancer Center Tuebingen-Stuttgart, Hoppe-Seyler-Str. 3, 72076 Tuebingen, Germany; 3 Department of Neurology and Interdisciplinary Neuro-Oncology, University Hospital Tuebingen, Hertie Institute for Clinical Brain Research, Hoppe-Seyler-Strasse 3, 72076 Tuebingen, Germany; 4 Department of Neuropathology, Institute of Pathology and Neuropathology, University Hospital Tuebingen, Calwerstr. 3, 72076 Tuebingen, Germany; 5 Department of Neurosurgery, University Hospital Tuebingen, Hoppe-Seyler-Str. 3, 72076 Tuebingen, Germany; 6 Diagnostic and Interventional Neuroradiology, Department of Radiology, University Hospital Tuebingen, Hoppe-Seyler-Str. 3, 72076 Tuebingen, Germany; 7 Department of Neurooncology, Department of Neurology, University Hospital Tuebingen, Hoppe-Seyler-Str. 3, 72076 Tuebingen, Germany; 8 German Cancer Consortium (DKTK) Partnersite Tuebingen, German Cancer Research Center (DKFZ), Heidelberg, Germany; 9 Clinic for Neurosurgery, Hospital Reutlingen, Reutlingen, Germany; 10 Department of Nuclear Medicine, University Hospital Tuebingen, Hoppe-Seyler-Str. 3, 72076 Tuebingen, Germany; 11 Department of Radiation Oncology, Eberhard-Karls-University of Tuebingen, Hoppe-Seyler-Str. 3, 72076 Tuebingen, Germany

**Keywords:** Atypical meningioma, Recurrence pattern analysis, MIB-1, Treatment planning, Additive radiotherapy, SSTR-PET/CT

## Abstract

**Background:**

Atypical meningiomas exhibit a high tendency for tumor recurrence even after multimodal therapy. Information regarding recurrence patterns after additive radiotherapy is scarce but could improve radiotherapy planning and therapy decision. We conducted an analysis of recurrence patterns with regard to target volumes and dose coverage assessing target volume definition and postulated areas of tumor re-growth origin. Prognostic factors contributing to relapse were evaluated.

**Methods:**

The clinical outcome of patients who had completed additive, somatostatin receptor (SSTR)-PET/CT-based fractionated intensity-modulated radiotherapy for atypical meningioma between 2007 and 2017 was analyzed. In case of tumor recurrence/progression, treatment planning was evaluated for coverage of the initial target volumes and the recurrent tumor tissue. We proposed a model evaluating the dose distribution in postulated areas of tumor re-growth origin. The median of proliferation marker MIB-1 was assessed as a prognostic factor for local progression and new distant tumor lesions.

**Results:**

Data from 31 patients who had received adjuvant (n = 11) or salvage radiotherapy (n = 20) were evaluated. Prescribed dose ranged from 54.0 to 60.0 Gy. Local control at five years was 67.9%. Analysis of treatment plans of the eight patients experiencing local failure proved sufficient extent of target volumes and coverage of the prescribed dose of at least 50.0 Gy as determined by mean dose, D98, D2, and equivalent uniform dose (EUD) of all initial target volumes, postulated growth-areas, and areas of recurrent tumor tissue. In all cases, local failure occurred in high-dose volumes. Tumors with a MIB-1 expression above the median (8%) showed a higher tendency for re-growth.

**Conclusions:**

The model showed adequate target volume and relative dose distribution but absolute dose appears lower in recurrent tumors without reaching statistical significance. This might provide a rationale for dose escalation studies. Biological factors such as MIB-1 might aid patients’ stratification for dose escalation.

**Supplementary Information:**

The online version contains supplementary material available at 10.1186/s13014-021-01825-2.

## Background

The current WHO classification of central nervous system tumors grades meningiomas depending on morphologic features, mitotic activity and brain invasion [1]. Atypical (WHO grade II) meningiomas exhibit a high tendency to relapse with up to 30–40% recurring or progressing within 5 years after gross total resection (GTR), posing a therapeutic challenge [2, 3]. Postoperative radiotherapy is evaluated routinely in interdisciplinary boards, depending on resection status and other clinical factors [4–7]. For tumors with risk factors for local failure adjuvant radiotherapy is advised, which is corroborated by the results of the prospective RTOG 0539 trial [8, 9]. Nevertheless, the benefits of adjuvant radiotherapy must be weighed against possible and long-term side effects and discussed openly with patients, some of which opt for a watch-and-wait approach. However, information on recurrence patterns following postoperative radiotherapy for atypical meningioma is scarce [10, 11] as patients comprise a heterogeneous group regarding previous treatments such as number of resections and the postoperative interval. Especially in this setting, recurrence pattern analysis is important to assess target volume extent and dose coverage and to identify regions likely contributing to tumor re-growth, regardless of previous treatments and risk factors, as has been demonstrated in other tumor entities [12]. In addition to other treatment approaches which are beyond the scope of this study such as stereotactic radiotherapy, systemic or targeted approaches, this could help to identify regions eligible for dose escalation in the future, possibly improving local control for patients receiving fractionated additional radiotherapy.

The aim of our study was to evaluate whether insufficient treatment volume extent or radiation dose distribution in the primary tumor and regions suspected of contributing to tumor re-growth are the main reasons for local tumor progression. Here, we analyzed the clinical outcome after additive postoperative radiotherapy for atypical meningiomas. For patients experiencing tumor progression, we examined radiation plans including volumes, dose, and coverage. This analysis was performed on both target volumes and postulated areas that might contribute to tumor re-growth (Volumes of Interest, VOIs). In addition, possible prognostic factors were assessed in an exploratory analysis.

## Methods

### Patient selection and characteristics

In this retrospective study, patients with histologically confirmed WHO grade II meningioma who underwent radiation therapy at our institution between October 2007 and October 2017 were included in the analysis. Most patients had been already reported in a different analysis on brain and bone invasion by Zwirner et al. [13]. Patients who had received stereotactic radiotherapy or radiosurgery were not included in this study. All patients were treated with high precision image-guided radiotherapy (IGRT) using cone-beam CT. The retrospective analysis of patients’ data and clinical outcome was approved by the university’s ethics committee (417/2017/B02). Patients had received either postoperative additive radiation therapy or salvage radiotherapy in case of recurrence after initial resection. Recurrence was defined as new tumor tissue after complete resection whereas progression was defined as growth of residual tumor tissue after incomplete resection as determined by neuroradiological assessment. Local and distant control was analyzed dependent on postoperative interval and MIB-1 expression (retrieved from neuro-pathological records available for routine clinical evaluation). MIB-1 was used as an antibody directed against Ki-67 as a marker for proliferation [14, 15]. Distant failure was defined as the occurrence of a second meningioma in follow-up imaging that was discontinuous with the index tumor (both within and outside the brain). An evaluation was also conducted regarding age, gender, number of surgical interventions, resection status as determined by Simpson grading, radiation dose, macroscopic tumor tissue at the start of radiotherapy, initial tumor symptoms, side effects, and dexamethasone medication during radiotherapy as well as MRI-findings and clinical examination results during follow-up. A first follow-up was conducted at 3 months after the end of treatment and then once a year for a period of at least 5 years (follow-up times were calculated from the first day of radiotherapy).

### Radiotherapy planning

**Fig. 1:**
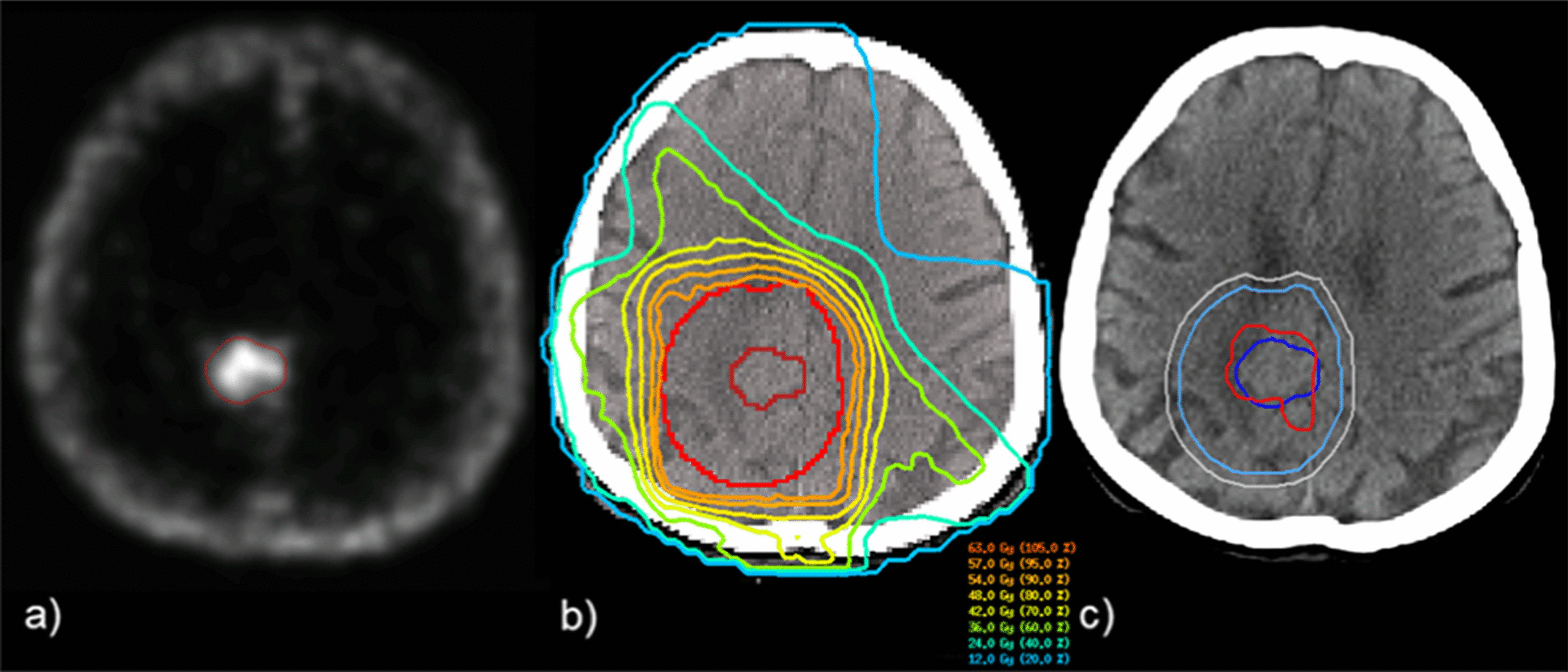
Clinical example for **a** SSTR-PET-CT imaging with high tracer uptake exhibited by the meningioma (red); **b** dose distribution as delivered during treatment (GTV (dark red), PTV (red)); **c** delineation of GTVini (blue) and GTVrt (red), CTV (light blue) and PTV (grey)

Radiotherapy was performed as normo-fractionated radiotherapy with fluence modulated radiotherapy (IMRT) and image guidance. Dose calculation was optimized using the inhouse planning system Hyperion [16, 17]. Dose prescription was a cumulative dose of 54.0–59.4/60.0 Gy in single fractions of 1.8–2.0 Gy at the discretion of the planning physician and did not vary between postoperative additive and salvage radiotherapy. A calculated dose reduction to the PTV was permitted depending on tumor localization and proximity to organs at risks (OARs). For treatment planning, target volumes were delineated on a planning CT scan (120 kV, 40 mAs, slice thickness 3 mm, field of view 600 mm, Somatom, Siemens) with fixation of the head using a thermoplastic mask. All patients had received recent MRI imaging (contrast enhanced T1w (T1 weighted, fat saturated), T2-FLAIR, slice thickness 3 mm) prior to treatment planning. MR imaging data were co-registered to the planning CT scans, using an automatic co-registration algorithm and manual adjustments, if needed. All patients underwent additional somatostatin-receptor-PET-CT (SSTR-PET-CT) imaging with either [68Ga]-DOTATATE or [68Ga]-DOMITATE. SSTR-PET-CT information was also co-registered for target volume definition. GTV was defined as the tumor-extent with uptake of contrast in T1 and/or as the resection cavity with contrast-positive residual tumor tissue. Dural tail was included as far as it showed tracer uptake in SSTR-PET-CT or if tumor infiltration was suspected in neuroradiological imaging. Target volume delineation was conducted with respect to OARs, comprising brain stem, optic nerves, chiasm, pituitary gland, eyes, lenses, lacrimal glands, cochleae (inner ear), and brain (detailed dose constraints are listed in Additional file 1: Table 1). The GTV was visually calibrated to the margins of existing tumor in T1w MRI to incorporate PET-positive areas at the primary tumor location or the resection cavity. The resection cavity was included in the GTV. The CTV was defined as the GTV with a 15 mm margin with respect to adjacent organs at risk and anatomical boundaries such as brain (5 mm except suspected brain invasion), falx, tentorium or skull. Around the CTV, a further 3–5 mm margin was added for the PTV (compare Fig. 1).

### Volume of Interest (VOI) analysis in case of local failure

**Fig. 2:**
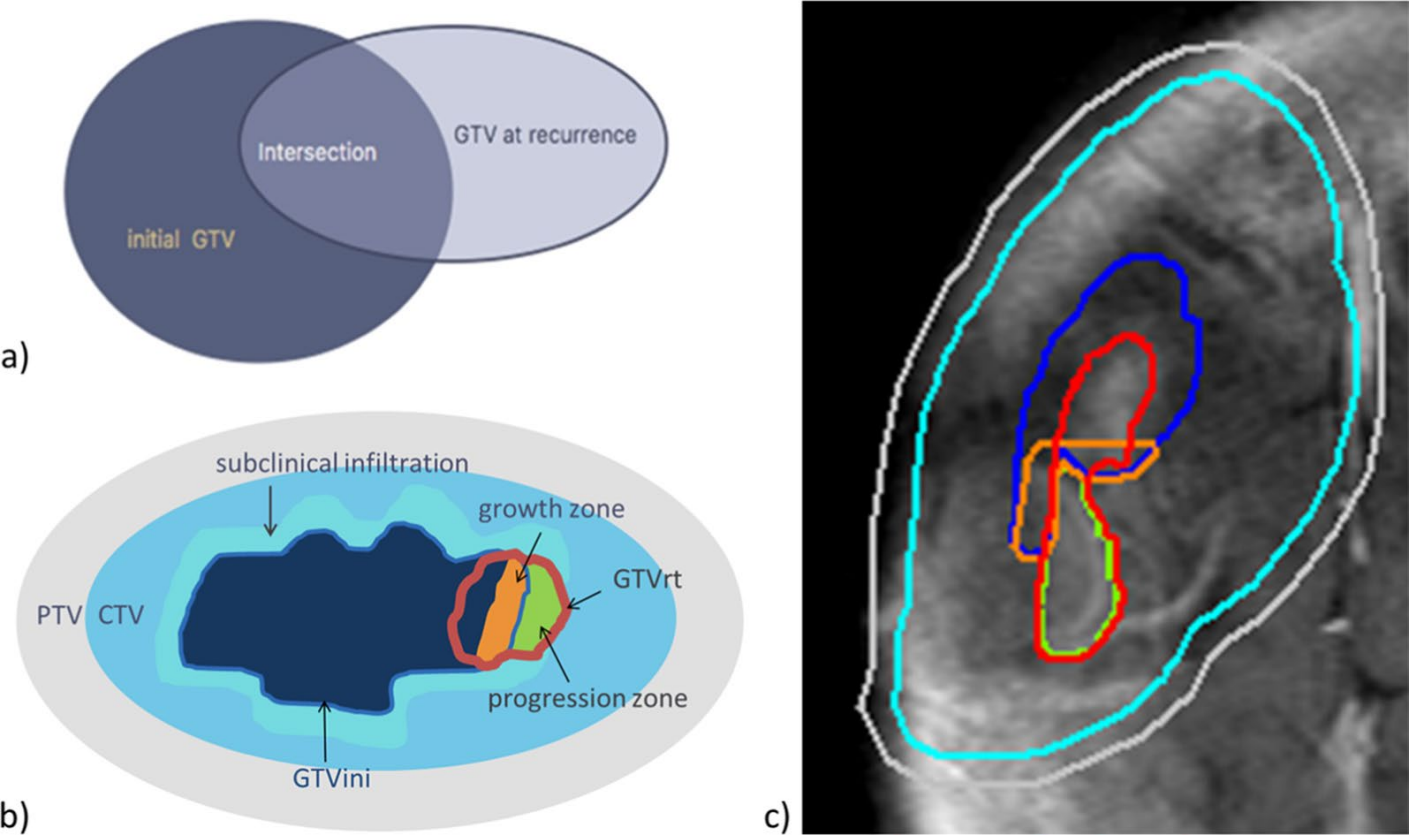
Schematic depiction of volumes. **a** Delineation of the initial GTV (GTVini), analysis volume GTV at recurrence/progression (GTVrt), and their intersection. **b** Schematic depiction of volumes. These volumes were postulated in order to identify regions within and adjacent to the initial GTV (GTVini, dark blue, dark blue outline) that might contribute to growth of recurrent tumor. GTV at recurrence (GTVrt, red margin) includes possible residual initial tumor tissue. Progression zone = new tissue without overlap with GTVini, green. Growth zone = 3 mm margin within the GTVini bordering on the progression zone (depicted in orange). Subclinical infiltration = GTVini + a 6 mm margin in all directions (depicted in turquoise). Initial CTV depicted in light blue, initial PTV in light grey. **c** Example of target volume and analysis volume evaluation in matched contrast enhanced T1 MR imaging showing recurrent tumor tissue, using colors corresponding to **b**. For clarity reasons, subclinical infiltration was omitted in this example

Contrast-enhanced MR-imaging was recorded for each follow-up. In the case of tumor recurrence/progression, dose coverage was assessed by co-registering MRIs with recurrent tumor tissue to the initial radiation planning CT scan and delineating recurrent tumor tissue as a separate analysis volume. With this approach, initial target volume delineation and dose volume histograms in the radiation treatment planning were analyzed with respect to the localization and presumed origin of the recurrent tumor in follow-up imaging, for which specific regions of interest were defined. For analysis, tumor recurrence and tumor progression were defined as new tumor tissue or continuous growth of the irradiated tumor, respectively, in follow-up contrast enhanced T1w MR-imaging as determined by neuroradiological assessment. For the analysis of tumor recurrence/progression, the first MRI that documented new tumor tissue or tumor growth was co-registered to the initial planning CT. In a first step, VOIs referring to the primary and recurrent tumor were defined (VOI definition listed in Additional file 1: Table 2). All tumor tissue in contrast enhanced T1w follow-up MR imaging was delineated as a separate analysis volume (“GTV at recurrence”, in the following GTV recurrent tissue; **GTVrt**). The intersection between the initial GTV (**GTVini**) prior to radiotherapy and GTVrt was defined as a separate analysis volume (“**Intersection**”; Fig. 2a). In order to gauge the extent of intersection between the GTVini and GTVrt, Dice‘s coefficient was calculated, describing the similarity of two intersecting volumes (Dice‘s coefficient = [2 x Intersection]/[GTVini + GTVrt]). In a second step, further volumes were defined in order to assess the dose coverage within the initial GTV (= GTVini) and the presumed subclinical infiltration around the tumor. To evaluate the dose distribution in the proximity of the GTVini, this subclinical infiltration zone was defined as a 6 mm margin around the GTVini (“**subclinical infiltration**”). All new tumor tissue in follow-up imaging was defined as a separate analysis volume, the “**progression zone**”. Furthermore, a growth zone was postulated, encompassing a 3 mm margin within the GTVini, adjacent to the progression zone (“**growth zone**”, a postulated zone within the GTVini from which progression might originate). For the different target and analysis volumes—GTVini, GTVrt, the progression zone, and growth zone (Fig. 2b)—dose volume histograms were calculated and analyzed for dose parameters (mean dose, D98, D2 and equivalent uniform dose (EUD: as the uniform dose that yields the same calculated biological effect, i.e. tumor control probability in this case, as the real non-uniform dose distribution)).

### Statistical analyses

For statistical analyses, SPSS was used (version 25, IBM Corp., Armonk, NY). A Pearson’s χ^2^-test was employed for comparison between groups using cross-classification tables. Local and distant intracerebral control was assessed by Kaplan-Meier analysis. Statistical evaluation of predictor variables of interest was performed using log rank tests. P-values and standard deviation are specified in each figure. Threshold for significance was set at p = 0.05 and at p = 0.10 for trend-level significance. For means standard deviations are detailed. When a median is reported, the range has also been specified. For box plot diagrams, an ANOVA-analysis was conducted.

## Results

Of the 33 patients included, 31 patients were eligible for statistical analysis of clinical outcome (local control vs. local recurrence) and possible prognostic clinical factors (Table 1). All patients had received at least one surgery prior to radiotherapy. The decision for adjuvant radiotherapy vs. salvage radiotherapy was based on the recommendation of the interdisciplinary tumor board and patient’s decision. In three cases (9.7%) static IMRT and in 13 patients dynamic IMRT was delivered, the other 15 patients were treated with VMAT. Eleven patients (35.5%) received adjuvant radiotherapy within 12 weeks after the last resection and 20 (64.5%) patients within one year postoperatively. The mean time between last surgery and radiotherapy was 1.67 years. Actual median follow-up was 4.39 years (range 0.13 to 10.45 years). Patients who suffered local tumor recurrence had a significant longer follow-up than those who did not experience local failure (6.68 years vs. 4.39 years, p = 0.028). Further analysis of patients’ characteristics, such as dexamethasone, gender, or age, did not yield any differences between groups (Table 1). In 13 patients (41.9%), no side effects were observed. Side effects were mostly mild (n = 9, 29%) and comprised dizziness, fatigue and mild concentration difficulties, with three patients (9.6%) reporting headaches. In two patients, seizures were observed, although one of these patients had reported seizures prior to treatment. However, in five patients, severe complications were encountered. Two patients experienced symptomatic radionecrosis and in one further case, radionecrosis was suspected in imaging. Two disease-related, but not radiotherapy-related, deaths occurred during the course of radiotherapy. Therefore, these two patients were eliminated from recurrence pattern analysis and statistical analysis for clinical and biological prognostic factors. One patient died due to complications of perforated diverticulitis and another patient with an underlying cardiological condition and symptomatic epilepsy died from cardiac arrest following a seizure.

**Table 1:** Patients’ characteristics

	Local control		Local recurrence		*P* value
	n = 23		n = 8		
*Age*					
At time of diagnosis [years]	64.0	*SD 13.2*	64.4	*11.0*	0.945
At start of radiotherapy [years]	66.2	*SD 12.8*	68.6	*9.5*	0.630
*Gender*					
Female/Male	12/11	*52.2%/47.8 %*	6/2	*75.0%/15.0 %*	0.260
*Prescribed dose*					
57.6/59.4/60.0 Gy	21	*91.3 %*	7	*87.5 %*	0.814
54.0 Gy	2	*8.7 %*	1	*12.5 %*	
*Postoperative interval < 12 weeks*					
< 12 weeks	9	*39.1 %*	2	*25 %*	*0.472*
> 12 weeks	14	*60.9 %*	6	*75 %*	
*Postoperative interval > 1 year*					
< 1 year	16	*69.6 %*	4	*50 %*	*0.319*
> 1 year	7	*30.4 %*	4	*50 %*	
*Postoperative interval [yrs]*	Median 0.28	*0.14–17.4*	Median 0.86	*0.16–2.43*	0.494
*Follow-up interval [yrs]*	Median 4.39	*0.13–10.45*	Median 6.68	*3.55–9.88*	0.028*
*Number of resections*					
Single resection	17	*73.9 %*	5	*62.5 %*	0.540
2 or more resections	6	*26.1 %*	3	*37.5 %*	
*Simpson Grading*					
I	6	*26.1 %*	0	*0 %*	0.315
II–III	9	*39.1 %*	2	*25%*	
IV–V	6	*26.1 %*	5	*62.5 %*	
Not detailed	2	*8.7 %*	1	*12.5 %*	
*Tumor tissue at start of radiotherapy*					
Macroscopic tumor present	14	*60.9 %*	7	*87.5 %*	0.208
No macroscopic tumor present	9	*39.1 %*	1	*12.5 %*	
*Tumor location*					
Frontal	13	*56.5 %*	1	*12.5 %*	0.081
Temporal	4	*17.4 %*	4	*75 %*	
Parietal	3	*13.0 %*	1	*12.5 %*	
Occipital	2	*8.7 %*	0	*0 %*	
Bordering on the skull base	1	*4.3 %*	2	*25 %*	
Accompanying symptoms**					
Presence of edema	3	*13.0 %*	3	*37.5 %*	0.245
Treatment with corticosteroids	12	*52.2 %*	4	*50.0 %*	0.916
Grading criteria**					
Brain invasion	10	*43.5 %*	3	*37.5 %*	0.768
Necrosis	5	*21.7 %*	3	*37.5 %*	0.380
High mitotic activity	6	*26.1 %*	2	*25.0 %*	0.952
Other criteria/not specified	5	*21.7 %*	2	25.0 %	0.849
*MIB-1*					
< Median	11	*47.8 %*	2	*25.0 %*	0.185
> Median	10	*43.5 %*	6	*75.0 %*	
Not detailed	2	*8.7 %*	0	*0 %*	
	Median 7 %	1–20 %	Median 10 %	4–20 %	0.358
*Target volume sizes [cm* ^3^ *]*					
GTV	Mean 36.15	*SD 40.53*	Mean 15.61	*SD 12.08*	0.173
PTV	Mean 163.02	*SD 75.32*	Mean 109.79	*SD 95.38*	0.119

Details of patients included in the analysis for factors preceding radiotherapy, n = 31. MIB-1 median was at 8%. For all categories except age, where a comparison of averages was conducted, a Pearson’s χ^2^ test was performed. Statistical threshold was set at p = 0.05, *statistically significant difference between groups, **multiple symptoms and grading criteria possible

Local control was achieved in 23 patients (74.2%). However, in three of these patients’ distant meningioma occurred. Eight patients experienced an in-field tumor progress or recurrence (25.8%). Of these, three patients also showed new distant tumor lesions outside the field and with no relation to the PTV. Two patients suffered a pathologically confirmed tumor progression to a more malignant phenotype of grade III. One patient developed pulmonary metastasis. Of the eight patients experiencing local failure, seven treatment plans were assessable for further evaluation.

**Fig. 3:**
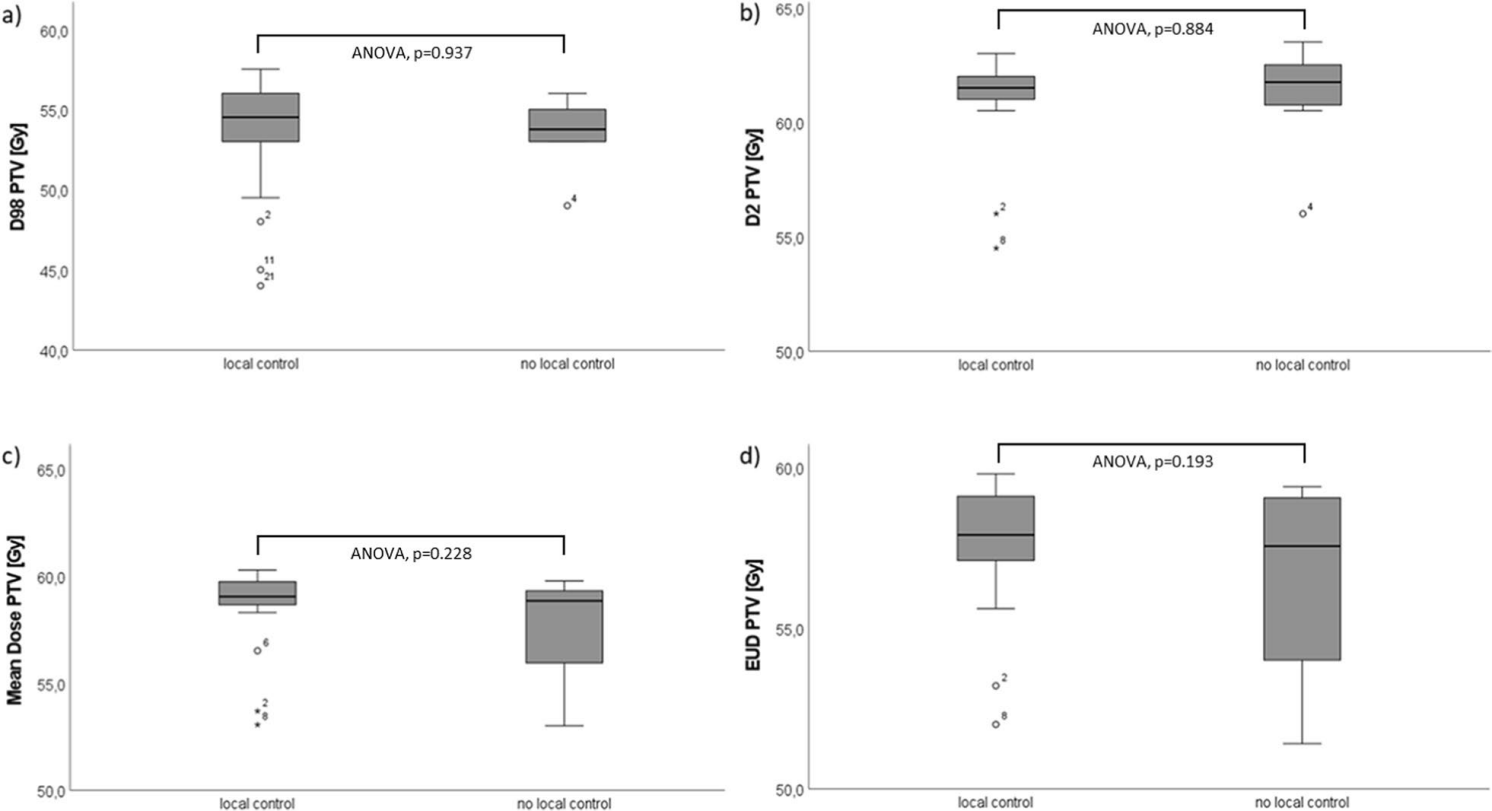
Box plot diagrams comparing the median, interquartile range, and range of **a** D98 of the PTV, **b** D2 of the PTV, **c** Mean dose of the PTV, and **d** EUD of the PTV between groups. All doses are reported in Gy. Boxes are reporting the interquartile range. Whiskers are indicating the range. Circles are referring to data points exceeding the range within 1.5 times the interquartile range. Stars are referring to data points exceeding 2.5 times the interquartile range

In all seven patients, initial target and analysis volumes—GTVini, GTVrt, progression zone, subclinical infiltration, and growth zone—were delineated as specified (detailed in a clinical example, Fig. 2c). For each target and analysis volume, extent was recorded (Table 2). Dice’s coefficient was 0.47 on average (SD  0.19; range 0.16–0.71), reflecting a generally high similarity and intersecting volume between the initial tumor volume and recurrent tumor volume (exception patient no. 2, who exhibited meningiomatosis with contralateral tumor in the first follow-up MRI scan). For all target and analysis volumes, DVHs (mean dose, D98, D2, and EUD) and differences in D98 between groups of patients experiencing relapse/tumor progression and of patient with local tumor control were assessed (Table 3). There was no significant difference between groups (i.e. patients with tumor recurrence vs. no tumor recurrence) regarding mean dose of the PTV, D98 or D2 of the analyzed volumes. Furthermore, there was no significant difference between groups regarding the EUD of the PTV (57.77 Gy in the group with no local recurrence, SD ± 2.02 Gy, 56.50 Gy in the group suffering from recurrence, SD ± 3.09 Gy, ANOVA p = 0.193). Dosimetrical data are detailed in Fig. 3 and Additional file 1: Table 3.

**Table 2:** Extent of target volumes in patients with tumor recurrence

ID	GTVini	GTVrt	Intersection	Progression zone	Growth zone	Dice’s coefficient
*1*	14.40	3.55	2.69	0.57	1.84	0.30
*2*	3.41	2.04	0.43	1.31	0.6	0.16
*3*	12.70	18.85	11.04	6.72	4.61	0.71
*4*	12.10	4.84	2.98	1.41	1.02	0.36
*5*	18.20	10.61	9.07	0.76	0.95	0.64
*6*	7.59	4.03	3.01	0.51	1.12	0.52
*7*	33.70	24.82	19.39	3.94	4.66	0.66

All volumes are given in cm^3^. Progression zone and Growth zone are referring to the size of the zone, defined as described in Fig. 2b). Dice’s coefficient was calculated using the volumes of GTVini, GTVrt, and their intersection, reflecting a high similarity in n = 6 cases. ID: patients’ number

**Table 3:** Dose coverage detailed in patients with tumor recurrence

ID	Prescribed dose	EUD PTV	D98 GTVini		D98 GTVrt		D98 progression		D98 growth zone	
			*Gy*	%	*Gy*	%	*Gy*	%	*Gy*	%
*1*	54.0	52.20	51.1	94.6	50.25	93.1	49.85	92.3	50.25	93.1
*2*	60.0	57.50	53.5	89.2	53.48	89.1	53.28	88.8	58.58	97.6
*3*	60.0	59.20	58.45	97.4	58.45	97.4	58.05	96.8	58.45	97.4
*4*	59.4	59.40	57.35	96.5	57.75	97.2	58.35	98.2	58.35	98.2
*5*	60.0	51.40	53.15	88.6	26.00	43.3	52.85	88.1	52.75	87.9
*6*	60.0	55.50	58.03	96.7	57.98	96.6	57.78	96.3	57.98	96.6
*7*	60.0	58.90	56.15	93.6	56.15	93.6	55.45	92.4	56.05	93.4

Prescribed doses and D98 values are detailed in Gy as well as in percent of the initially described dose. One patient (no. 5) suffered an extended tumor relapse with meningiomatosis and affection of the contralateral meninges, resulting in comparably low dose coverage of GTVrt. ID: patients’ number

**Fig. 4:**
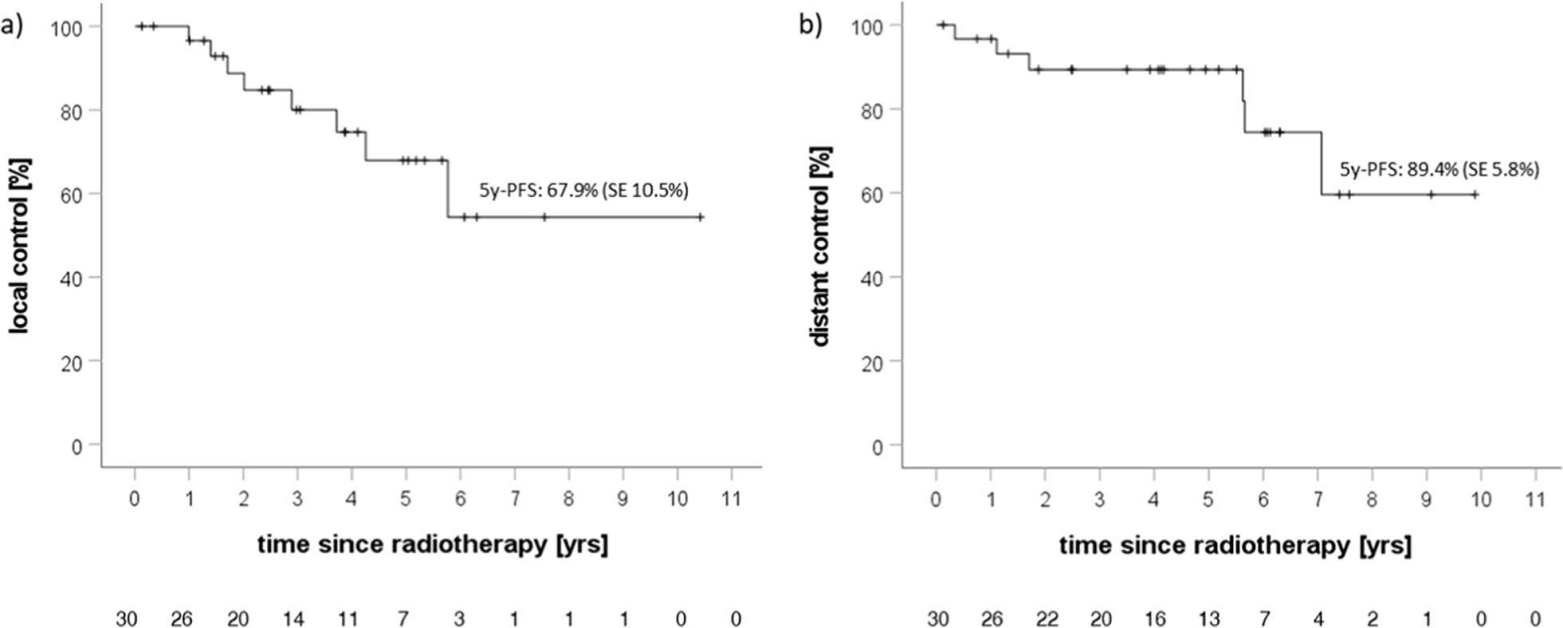
Kaplan-Meier curves regarding **a** local control and **b** distant control. Three patients experienced both local failure and new distant lesions. Time interval refers to time since start of radiotherapy. Number of patients at risk is detailed below the corresponding curves

**Fig. 5:**
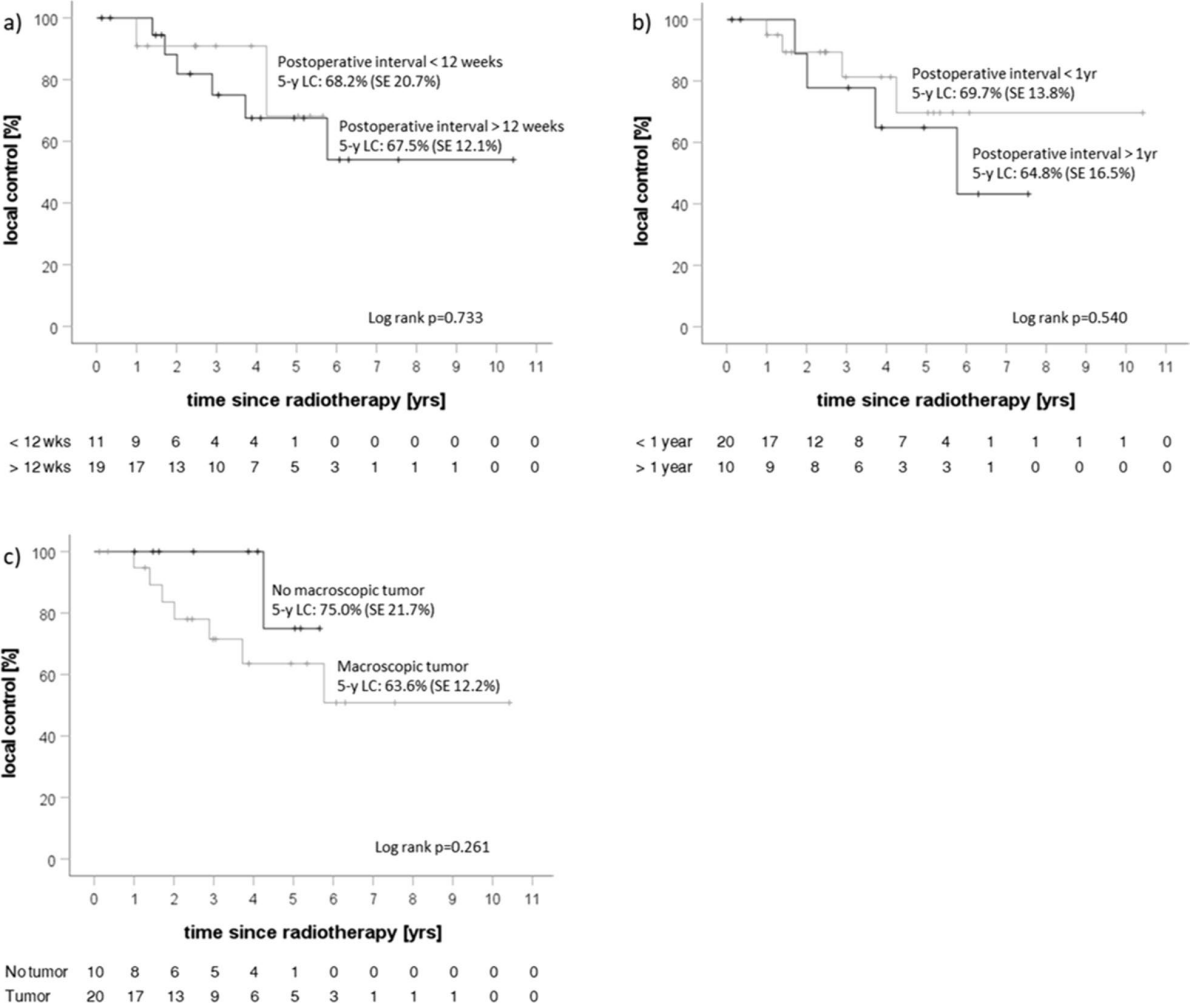
Kaplan-Meier curves regarding **a** local control with regard to the interval between last tumor resection and start of radiotherapy with a postoperative interval of 12 weeks; **b** local control with regard to the interval between last tumor resection and start of radiotherapy with a postoperative interval of 1 year; **c** local control with regard to macroscopic tumor before the start of radiotherapy. Time interval refers to time since start of radiotherapy. Log rank values refer to the whole observation period. Number of patients at risk detailed below the corresponding curves

**Fig. 6:**
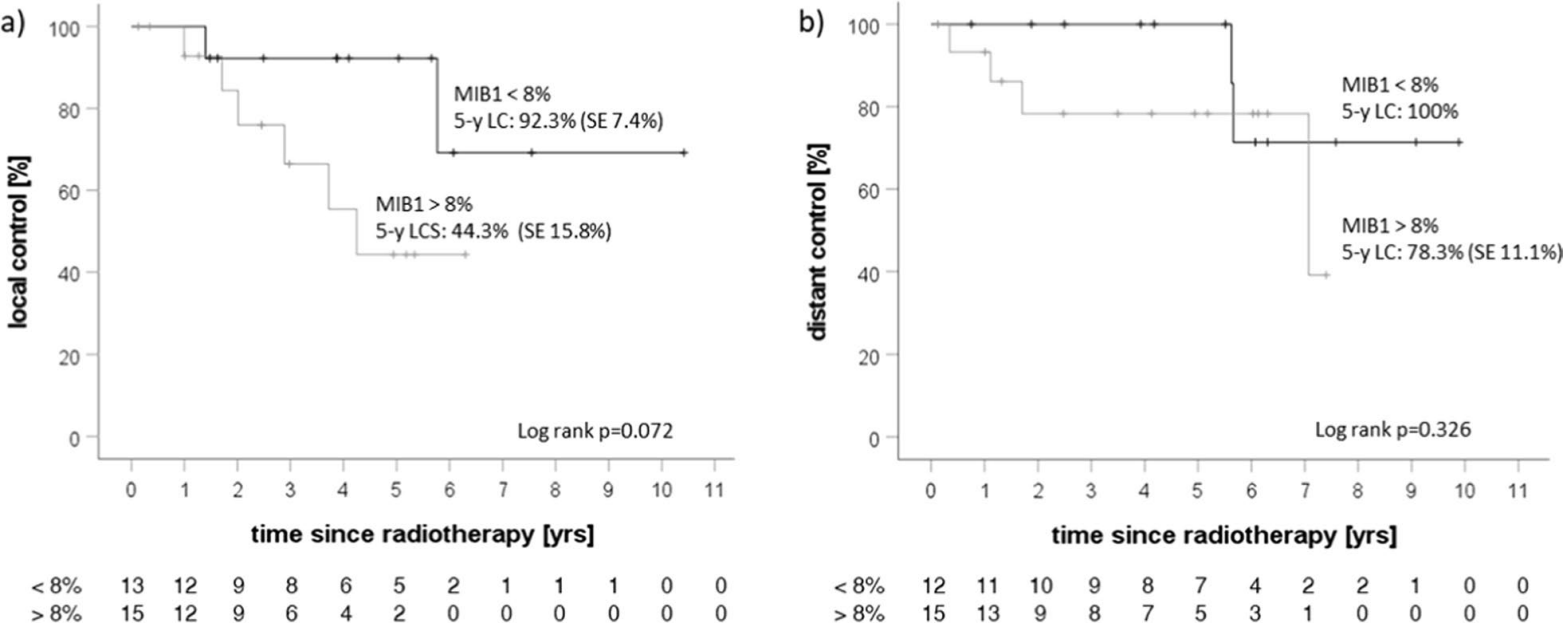
Kaplan-Meier curves depicting **a** local control with regard to MIB-1 proliferation index [median 8%]; **b** distant intracerebral control with regard to MIB-1 proliferation index, as determined in the histology of the tumor lesion that received postoperative radiotherapy. Time interval refers to time since start of radiotherapy. Log rank values refer to the whole observation period. Number of patients at risk is detailed below the corresponding curves

Local and distant control were analyzed (Fig. 4a, b) and compared with regard to exploratory prognostic factors. Local control was 67.9% at 5 years. In addition to local control for the whole cohort, local control with regard to macroscopic tumor before initiation of radiotherapy (Fig. 5c) was analyzed (5-y local control of 75% without macroscopic tumor vs. 63.6% for patients with macroscopic tumor tissue, p = 0.261). There was no significant difference in progression-free survival between groups for both a postoperative interval of 12 weeks and one year (Fig. 5a, b). MIB-1 proliferation index was assessed and associated with local control at 5 years, which showed local control in 92.3% of patients with a MIB-1 below the median of 8% vs. 44.3% for patients with MIB-1 expression above the median (p = 0.072, Fig. 6a). Occurrence of new distant tumor lesions was not associated with MIB-1 expression (Fig. 6b).

## Discussion

Atypical meningiomas exhibit a tendency to relapse and a significant number of patients suffer from tumor recurrence after radiotherapy, but there is limited literature concerning spatial patterns of tumor recurrence following additive radiotherapy. Recurrence patterns have been evaluated for gliomas [18, 19], but information on recurrence patterns in atypical meningioma after fractionated radiotherapy remains scarce. Zollner et al. analyzed local control in high-grade meningioma after PET-based treatment planning with regard to safety margins [10] and Rajkrishna et al. evaluated recurrence patterns after conformal radiotherapy, but in a group of patients with both low-grade and high-grade meningioma [11]. As patients with atypical meningioma comprise a heterogeneous group regarding prior treatments, we conducted a recurrence pattern analysis with respect to initial target volumes and, in a novel approach, to regions as proposed origin of tumor re-growth by postulating a growth zone, progression zone, and subclinical infiltration zone. Our analytical approach proved viable by using co-registration of follow-up MRI scans with initial imaging, reliable definition of target and analysis volumes, and robust identification of regions likely contributing to tumor recurrence.

### Study approach

In our retrospective analysis of recurrence patterns after additive radiotherapy in atypical meningioma, the proposed model allowed local recurrence analysis. Target volume definition and dose coverage with regard to recurrent tumor tissue was conducted and showed local tumor relapse in 25.8%, not associated with dosimetrical misses with regard to the initial target volumes. Exploratory prognostic factors were evaluated and showed an association, however not significant, between tumor relapse and proliferative activity (as measured by MIB-1 expression determined in neuropathology specimen).

### Dosimetrical analysis

In all patients with local relapse analyzed in our study, tumor re-growth occurred in the PTV. This is corresponding to other analyses that found mostly in-field local failure [10]. In six patients (22.6%), new distant and separate tumor lesions occurred during follow-up. This is in line with studies that reported a median of 3.1 tumor lesions occurring in patients with multiple meningiomas [20]. In case of local recurrence, the initial target volumes, proposed growth areas, and the recurrent tumor tissue received a dose within the ICRU50 prescription of 60.0 Gy in 3/7 cases whereas also the other 4/7 received at least 50.0 Gy, owing to a lower prescribed dose of 54.0 Gy or OARs and corresponding to 88% of a prescribed dose of 60.0 Gy [21]. Margin definition for CTV and PTV in our cohort was in line with recommendations for extended margins in atypical meningioma with a recommended CTV margin of 15 mm around the GTV [10, 22]. In all patients with relapse, the growth zone was covered by the initial PTV and received a minimal dose of 50.25 Gy and the PTV received an EUD of at least 51.40 Gy. No significant difference between both groups could be observed regarding D2, D98, mean dose, and EUD of the PTV. Local failure occurred despite optimized treatment planning using SSTR-PET/CT, which helps to discern tumor tissue from postoperative alterations and has been shown to optimize target volume delineation [4, 23]. This does not indicate a dosimetrical miss in dose delivery resulting in recurrent meningioma growth while showing sufficient extent of target volumes and safety margins. Analysis of Dice’s coefficient showed a high similarity and thus, a significant overlap between initial tumor volume and recurrent tumor volume. As dose coverage of target volumes was sufficient, it might be hypothesized that for improving local control in atypical meningioma, doses should at least reach 60.0 Gy. High doses attained with proton therapy [24] and in stereotactic radiotherapy are corroborating this hypothesis [25, 26], but especially large tumors and resection cavities might not be eligible for stereotactic radiotherapy. However, in the interpretation of our study results it should be considered that additive radiotherapy for atypical meningioma was conducted restrainedly - postoperative therapy decisions at our institution often favored a watch-and-wait approach with initiation of radiotherapy in case of tumor progression, resulting in a negative bias regarding our patient collective. The application of comparable doses, especially in normofractionated photon radiotherapy, however, to larger target volumes within or in proximity to the brain needs to be weighed against an increased risk of radionecrosis [27]. An identification of a smaller possible boost-volume within the resection cavity or residual tumor tissue, respectively, would be desirable, but in our retrospective study no specific area as origin of tumor recurrence could be established. The EORTC 22042–26042 trial is addressing the issue of dose escalation by investigating adjuvant high-dose radiotherapy for high-grade meningioma with initiation of postoperative radiotherapy within 6 weeks and administration of 60.0 Gy for gross-totally resected and 70.0 Gy for subtotally resected meningioma. Local control was improved for patients with gross-total tumor resection and an adjuvant dose of 60.0 Gy, but the outcome of patients with subtotal resection who received an adjuvant therapy with 70.0 Gy has not yet been published [28]. Therefore, a dose of 60.0 Gy might be sufficient in case of gross total resection, but the significance of dose escalation in normo-fractionated postoperative radiotherapy in case of residual macroscopic tumor has yet to be evaluated.

### Analyses of possible prognostic factors

In addition to the analysis of recurrence patterns, we evaluated several clinical and tumor-specific prognostic factors. In case of no macroscopic residual tumor before initiation of radiotherapy, tumor recurrence occurred less often and later than in patients with macroscopic tumor tissue (5-y local control 75% vs. 5-y local control 63.6%, p = 0.261, Fig. 5c). Macroscopic tumor is a known risk factor for tumor recurrence. In the RTOG 0539 trial, patients with residual or recurrent tumor were treated in the high-risk group, receiving 54.0 Gy with an integrated boost with 60.0 Gy to the macroscopic tumor [9], reflecting the dose prescription in our group of patients. A sufficient dose of at least 60.0 Gy in the macroscopic tumor seems necessary as in our cohort of recurrences, GTVini dose was below 95% of 60 Gy in 4/7 patients (Table 3). However, there was no systematic difference in the D98 of the GTVini and the D98 in the growth or progression zone between patients with local control and local failure. Apparently, although there was no difference between groups regarding the size and mean dose of both the PTV and GTV between groups, it cannot be excluded that already small compromises of the dosage to the GTV might favor tumor recurrence. Although a trend for likelihood of local tumor recurrence could be observed when macroscopic tumor was present before start of radiotherapy, in our log-rank analysis, results did not reach statistical significance due to small sample size.

We also analyzed the length of the postoperative interval as a prognostic factor. The length of the adjuvant interval is often not specified and definitions range from 6 weeks [8, 9] to 1 year [29]. However, no correlation between the likelihood for local failure and the length of the postoperative interval could be found, neither for a postoperative interval of twelve weeks nor for an interval of one year (p = 0.773 and p = 0.540, respectively). The significant longer follow-up in the group experiencing local failure (p = 0.028) was most likely owed to consecutive therapeutic interventions such as ligand-therapy or re-irradiation and further follow-up thereafter. However, a longer follow-up is needed as meningioma can recur several years after initial treatment.

Furthermore, MIB-1 index was investigated as a biological factor known to be correlated with tumor recurrence in meningioma and tumor grading [30–32]. As a marker for cellular proliferation, it is often detailed in histology reports but currently not included explicitly as a criterion for tumor grading in the WHO classification [1]. Chen et al. could show that patients with a MIB-1 index > 7% were at higher risk of tumor recurrence, irrespective of resection status [30]. In our cohort, we found a trend-level significant correlation between recurrence of tumor and a high MIB-1 index as a tumor-specific biological factor. The median MIB-1-index in our cohort of atypical meningiomas was 8% and local control tended to be associated with a MIB-1 index above the median (not significantly, however; 5-y local control of 44.3% vs. 92.3%, p = 0.072 in log-rank test). Although the association between a high MIB-1-expression and likelihood of local tumor recurrence needs to be interpreted with caution, we hypothesize that a high MIB-1-index might indicate a more aggressive tumor biology with a higher probability for tumor relapse, warranting a more offensive treatment approach as well as a more frequent and longer follow-up in patients exhibiting a high MIB-1 index.

### Study limitations

As our study cohort consisted of a small number of patients with a complex prior medical history and was conducted retrospectively, the prognostic value of MIB-1 regarding local and distant progression needs to be interpreted with caution. Furthermore, the retrieval of MIB-1 expression from pathological files is an estimate and may vary between pathologists. Also, due to the small number of patients, there were few cases of tumor recurrence, limiting interpretations derived from our analysis. However, our analysis was based on pathological records used in interdisciplinary boards and thus reflects the clinical setting. Whether the expression of MIB-1 could be a reliable prognostic factor and could aid therapy decision regarding dose escalation and additive radiotherapy should therefore be evaluated in a prospective study in a larger patient cohort and a long-term follow-up.

## Conclusions

In the light of the different factors contributing to the complex course of the disease and the divergent results of retrospective studies regarding the benefit of additive radiotherapy, some researchers have proposed patient stratification according to risk groups, taking into account the heterogeneity of tumor biology and tumor behavior within the group of atypical meningioma [33, 30], suggesting that the atypical tumor grading alone might not suffice when contemplating additive radiotherapy. In future analyses, a comparison with a similar group of patients who do not receive additive radiotherapy could further elucidate the value of additive radiotherapy regarding local control and the prognostic value of MIB-1. Also, our analysis and the proposed model for analyzing recurrence patterns underscores the necessity of reaching high doses in the macroscopic tumor in order to improve tumor control. Taken together, clinical and especially biological prognostic factors like MIB-1 could help identify patients with a high risk of tumor relapse who might benefit from early and intensified adjuvant radiotherapy as well as from dose escalation of macroscopic tumor tissue.

## Summary

As atypical meningiomas show a tendency to relapse despite additive radiotherapy, there is a need for identification of prognostic factors and optimization of treatment planning. In our patient cohort, tumor relapse and progression occurred in 25.8% of patients despite optimized treatment planning using SSTR-PET-CT, sufficient extent of target volumes and adequate relative dose coverage. Apparent dosimetrical misses could not be observed. Distinct areas within the tumor that received an insufficient dose coverage and are thus suspected to have contributed to tumor regrowth and could benefit from a possible dose-escalation, could not be identified in our small cohort exhibiting local failure with prescribed doses of 54.0–60.0 Gy. In line with findings from other studies, we found that a high MIB-1 index and macroscopic tumor at the start of radiotherapy was correlated, however not statistically significant, with a higher risk for tumor relapse. A longer postoperative interval of one year was not correlated with a higher risk for local failure. More research is needed to evaluate to which extent a dose escalation beyond 60.0 Gy can improve local control in atypical meningioma. Furthermore, routine assessment of clinical and biological prognostic factors could aid patient stratification regarding additive radiotherapy in the future.

## Supplementary Information

**Additional file 1**. **Table 1.** Dose constraints for Organs at Risk (OARs). **Table 2.** Volume of interest (VOI) definition. **Table 3.** Statistical information for the comparison of dosimetrical data. 

## Data Availability

The datasets analyzed during the current study are available from the corresponding author on reasonable request.

## Abbreviations

CT: Computed tomography
CTV: Clinical target volume
DVH: Dose volume histogram
EUD: Equivalent uniform dose
GTR: Gross total resection
GTV: Gross tumor volume
GTVini: Initial GTV
GTVrt: GTV at recurrence
ICRU: International Commission on Radiation Units
IMRT: Intensity-modulated radiotherapy
MIB-1: Molecular immunology Borstel
MRI: Magnet resonance imaging
OAR: Organs at risk
PFS: Progression-free survival
PTV: Planning target volume
SSTR-PET-CT: Somatostatin receptor PET-CT
SU: Standardized uptake
T1w: T1 weighted
VMAT: Volumetric arc therapy
VOI: Volume of Interest
WHO: World Health Organization
