# Supplementary material for: Retrospective analysis of recurrence patterns and clinical outcome of grade II meningiomas following postoperative radiotherapy
Source: Radiat Oncol. 2021 Jun 25;16:116. doi: 10.1186/s13014-021-01825-2 (PMC8235826; doi:10.1186/s13014-021-01825-2)
Supplement: Supplementary file 1 — Additional file 1. Table 1. Dose constraints for Organs at Risk (OARs). Table 2. Volume of interest (VOI) definition. Table 3. Statistical information for the comparison of dosimetrical data. [file 13014_2021_1825_MOESM1_ESM.docx]

**Supplementary Tables**

**Supplementary Table 1. Dose constraints for Organs at Risk (OARs).**

| *Organ at risk* |  | *Dose constraint* |
| --- | --- | --- |
| Brain stem |  | D max ≤ 54 Gy |
| Optic chiasm |  | D max ≤ 54 Gy |
| Optic nerve |  | D max ≤ 54 Gy |
| Brain (without PTV) |  | V40 < 10% |
| Lacrimal gland |  | Median < 40 Gy |
| Retina |  | D2% < 40 Gy |
| Lens |  | D max < 6 Gy |
| Inner ear |  | Mean < 45 Gy |

**Supplementary Table 2. Volume of interest (VOI) definition.**

| *VOI name* | *VOI definition* |
| --- | --- |
| GTVini | initial GTV, encompassing resection cavity, residual tumor tissue, and clearly thickened dural tail |
| GTVrt | GTV at recurrence, encompassing all tumor tissue; encompassing residual tumor tissue after completion of radiotherapy, growth zone, and progression zone in the first MRI imaging showing progress or recurrence |
| CTV | 15 mm margin surrounding the GTV |
| PTV | 3-5 mm margin surrounding the CTV |
| Growth zone | postulated zone, from which progression might originate, encompassing a 3 mm margin within the GTVini, adjacent to the progression zone |
| Progression zone | new tumor tissue in the first follow-up imaging showing tumor progress or recurrence outside GTVini |
| Subclinical infiltration | postulated zone of subclinical infiltration around the tumor; defined as a 6 mm margin around the GTVini |

VOI – volume of interest, GTV – gross tumor volume, CTV – clinical target volume, PTV – planning target volume.

**Supplementary Table 3. Statistical information for the comparison of dosimetrical data.**

|  | | *EUD PTV* | *Mean Dose PTV* | *D98 PTV* | *D2 PTV* |
| --- | --- | --- | --- | --- | --- |
| *Local control* | Mean | 57.77 | 58.68 | 53.67 | 61.07 |
|  | N | 23 | 23 | 23 | 23 |
|  | SD | 2.02 | 1.87 | 3.68 | 1.92 |
|  | Minimum | 52.00 | 53.05 | 44.0 | 54.50 |
|  | Maximum | 59.80 | 60.27 | 57.50 | 62.50 |
|  | Median | 58.40 | 59.04 | 55.00 | 61.50 |
|  |  |  |  |  |  |
| *No local control* | Mean | 56.50 | 57.62 | 53.56 | 61.19 |
|  | N | 8 | 8 | 8 | 8 |
|  | SD | 3.09 | 2.68 | 2.19 | 2.31 |
|  | Minimum | 51.40 | 53.00 | 49.00 | 56.00 |
|  | Maximum | 59.40 | 59.76 | 56.00 | 63.50 |
|  | Median | 57.55 | 58.84 | 53.75 | 61.75 |

All doses are specified in Gy. SD – standard deviation; EUD – equivalent uniform dose; PTV – planning target volume. N - number of patients.
